# Delayed Coronary Artery Perforation of a Nontarget Distal Small Side Branch With Subsequent Cardiac Tamponade After Percutaneous Coronary Intervention

**DOI:** 10.1155/cric/6234818

**Published:** 2025-11-11

**Authors:** Masaki Fujiwara, Masao Takemoto, Shintaro Umemoto, Yoshibumi Antoku, Takuya Tsuchihashi

**Affiliations:** Cardiovascular Centre, Social Medical Corporation, Steel Memorial Yawata Hospital (Seitetsu Kinen Yawata Byoin), Kitakyushu, Japan

**Keywords:** coronary artery disease, delayed coronary perforation, guide wire, percutaneous coronary intervention, pericardiocentesis, tamponade

## Abstract

Coronary artery perforations (CAPs) during percutaneous coronary interventions (PCIs) are rare and potentially fatal complications. Delayed CAPs are extremely rare. Here, we report a case of a delayed CAP in a small side branch that was not the target vessel of the PCI, which was difficult to salvage and was caused by a hydrophilic guidewire during PCI for bifurcation lesions. Finally, intracoronary administration of gelatin microspheres resulted in complete occlusion of the CAP site. Although the mechanism(s) of the delayed CAP onset had not been entirely elucidated, its pathogenesis is believed to have been multifocal. The key factors contributing to the delayed CAP may have included the nimious platelet-suppressing effects of dual-antiplatelet therapy, the prolonged anticoagulant effect of heparin used during PCI, the use of stiff-tip and hydrophilic guidewires, inadvertent guidewire advancement into small coronary arteries, and the fragility of coronary arteries associated with coronary risk factors. Meticulous attention to any patient signs, symptoms, or a nimble definite diagnosis, and effective, timely management of delayed CAPs may be essential for practicing cardiologists to reduce subsequent complications and improve patient prognosis after PCI, especially in cases of complex lesions.

## 1. Introduction

Coronary artery perforations (CAPs) during percutaneous coronary intervention (PCI) are rare complications but can result in cardiac tamponade and are thus potentially life-threatening [1–3]. It is also well recognized that the hydrophilic coronary guidewires used during PCI increase the risk of CAPs [2, 4]. CAPs mostly occur during the procedure, immediately after stenting, or adjunctive ballooning [2, 3], but delayed CAPs are extremely rare [4]. Herein, we report a case of a delayed CAP in a small side branch that was a nontarget vessel for PCI, which was difficult to salvage and was caused by a hydrophilic guidewire used during PCI for bifurcation lesions.

## 2. Case Report

**Figure 1:**
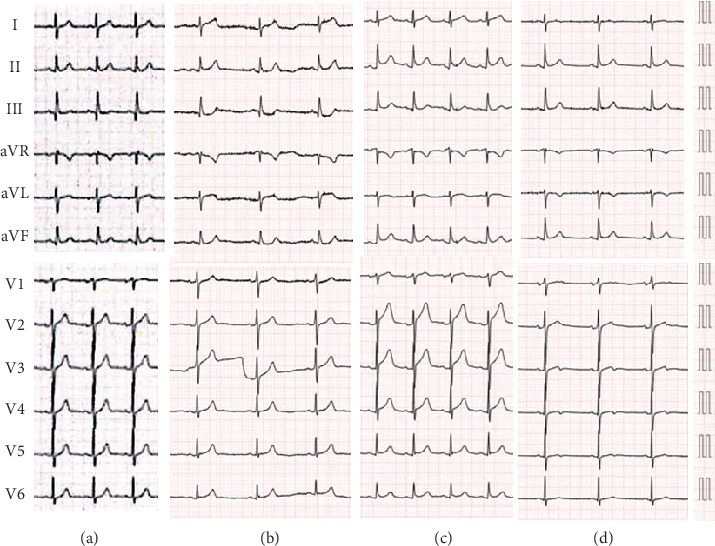
Twelve-lead electrocardiogram findings on (a) admission, at the (b) onset of a delayed coronary artery perforation (CAP) and (c) acute pericarditis, and (d) after treatment of the delayed CAP.

**Figure 2:**
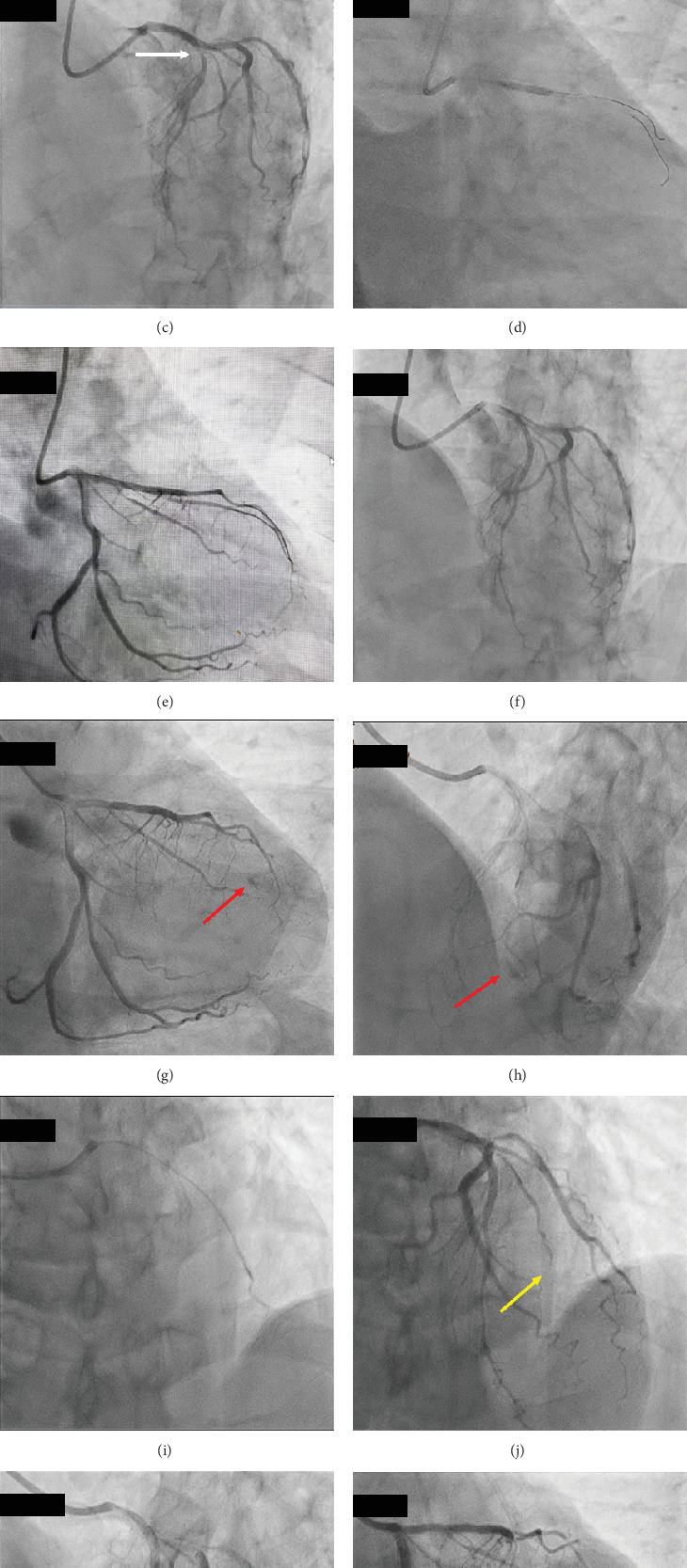
Coronary angiography of the (a) right and (b–l) left coronaries (a–c) before and (d–f) after percutaneous coronary intervention (PCI) and (g,h) during a delayed coronary artery perforation (CAP), (i) injection of gelatin microspheres, and (j–l) after complete occlusion of the delayed CAP. The white arrows in (b) and (c) indicate PCI target sites, red arrows in (g) and (h) indicate the site of the delayed CAP, and yellow arrows in (j–l) indicate the site of the complete occlusion of the delayed CAP.

**Figure 3:**
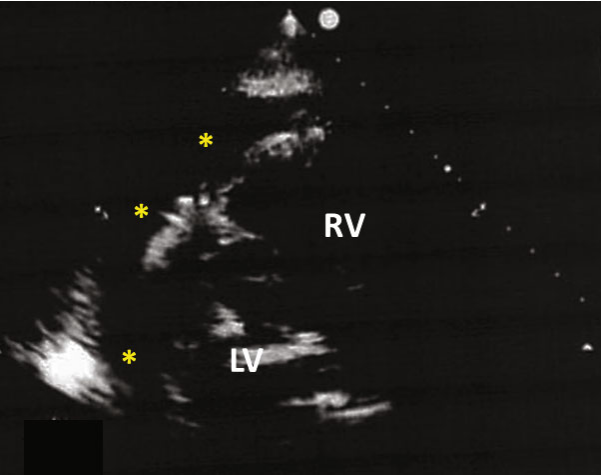
Echocardiography findings of a substantial pericardial effusion (yellow asterisks). LV = left ventricle, RV = right ventricle.

A 73-year-old male was admitted to our hospital with an increased number of episodes of chest discomfort on exertion. Coronary risk factors included hypertension, Type 2 diabetes mellitus, and dyslipidemia. He was an ex-smoker and nonalcohol drinker. He was taking medications, including candesartan 2 mg, carvedilol 10 mg, and rosuvastatin 2.5 mg. His blood pressure was 126/66 mmHg, with a pulse rate of 60 beats/min and regular rhythm. His HbA1c and low- and high-density lipoprotein cholesterol levels were 6.8% and 128 and 52 mg/dL, respectively. Both the electrocardiogram (Figure 1a) and echocardiogram on admission were normal. Coronary angiography (CAG) revealed a normal right coronary artery (Figure 2a) and 75% bifurcation stenosis of the proximal left anterior descending (LAD) coronary artery, with a fractionated flow reserve of 0.74, as well as 75% proximal stenosis of the diagonal branch (DB) of the left coronary artery (white arrows in Figure 2b,c). PCI via a right radial artery approach was performed after a 2-week oral administration of aspirin 100 mg and clopidogrel 75 mg. Following an intravenous bolus injection of heparin of 8000 IU (140 IU/kg body weight), a 6-Fr Launcher EBU3.5 guiding catheter (Medtronic) was positioned in the ostium of the left main trunk. A hydrophilic guidewire (Sion; ASAHI INTECC) was advanced into the LAD, and a second hydrophilic guidewire (Runthrough Floppy; Terumo) was positioned to protect the DB. After inflating a 2 × 10 mm balloon catheter (BC) (ZINRAI; Kaneka Medical) at the stenosis of the LAD, a plaque extension with no calcification was confirmed with an AltaView intravascular ultrasound catheter (IVUS) (Terumo). The proximal LAD lesion was then treated with a 2.5 × 15-mm sirolimus-eluting stent (Ultimaster Nagomi, Terumo) (Figure 2d). Since IVUS revealed incomplete stent dilation at the proximal stent region, a 2.75 × 8-mm Pantera Leo BC (Biotronik) was inflated at a high pressure of 14 atm. Subsequently, the coronary flow in the DB was unrestricted. IVUS revealed complete stent dilation, and the final CAG demonstrated satisfactory angiographic results with no contrast enhancement with extravasation and/or oozing (Figure 2e,f and Supporting Information 1 and 2). The procedure took 48 min. When he came to the ward, he was stable and his blood pressure was 118/64 mmHg. However, 2 h after PCI, he experienced chest pain with mild ST elevation in Leads I and aVL and ST depression in Leads III and aVF on the electrocardiogram (Figure 1b). His blood pressure dropped to 92/68 mmHg, and pulse rate was 53 beats/min, regular. Thus, intravenous administration of high-dose saline was initiated. Emergency CAG revealed an Ellis Type IIIa CAP [5] in a small distal branch of the DB (red arrows in Figure 2g,h and Supporting Information 3 and 4), a nontarget PCI vessel, accompanied by worsening hemodynamics with a blood pressure of 74/56 mmHg. Echocardiography revealed a substantial pericardial effusion (Figure 3), prompting immediate pericardiocentesis and placement of a pericardial drain. The evacuation of 400 mL of minimally blood-stained fluid promptly reverted his clinical status. A 2.0 × 10-mm ZINRAI BC was then delivered to the distal site of the DB and inflated at a low pressure of 3 atm for 30 min. Then, the activated clotting time (ACT) was 158 s. However, the perforations were not repaired. We then injected an autologous blood clot through a microcatheter (Caravel MC; ASAHI INTECC) by stirring blood collected from the sheath, but the perforation persisted. Finally, we dissolved 120 mg of gelatin microsphere [6] (Serescue; Astellas) in 10 mL of contrast media and injected 0.2 mL (2.4 mg of Serescue) into the DB coronary arteries via the microcatheter (Figure 2i and Supporting Information 5). The perforated distal site of the DB was completely occluded (yellow arrows in Figures 2j, 2k, and 2l and Supporting Information 6 and 7). After 60 min of observation, contrast injection showed a completely sealed perforation. Postprocedure laboratory assessment revealed a creatinine phosphokinase (CK) level of 778 IU/L and CK-MB isozyme of 95.6 IU/L. Thirty hours after the procedure, the patient complained of severe chest pain with ST elevation in precordial Leads V1∼4 on the electrocardiogram (Figure 1c). Laboratory data revealed no reascension of the cardiac enzymes (CK = 443 IU/L). CAG showed negative results including CAPs and stent thrombosis, leading to diagnosis of acute pericarditis due to the CAP. The patient was discharged on Day 11 after PCI, following treatment for acute pericarditis. His electrocardiogram on discharge was almost normal except terminal T wave inversion in precordial Leads V3∼5 (Figure 1d). He has remained well without any symptoms for 2 years post event.

## 3. Discussion

The incidence of CAP-associated PCI is generally 0.4% but can reach 4.1% during complex procedures [3]. Some patients develop severe complications, such as cardiac tamponade and even death [1, 2, 7]. One-year mortality after a CAP is 2–3 times higher than that in patients without CAP [2, 3]. Immediate successful pericardiocentesis for cardiac tamponade due to CAPs may be the cornerstone for saving a patient's life. According to the Ellis classification [5], a mild CAP (Ellis Types I and II) generally has a better prognosis and can often be managed conservatively, with most patients undergoing prolonged balloon inflation alone. In more severe cases (Ellis Type III), the in-hospital mortality rate is very high (15.2%), and these patients require urgent treatment [7]. Recent increases in the availability and use of stiff-tip and hydrophilic guidewires may have increased both the potential risk and severity of CAPs [2, 3]. Therefore, when using hydrophilic guidewires, especially in complex lesions, the operators should be cautious of the potential for CAPs. Treatment options include covered stents, coils [2], or other methods such as autologous blood clots [8], thrombin, fat, or microsphere injection [2], with surgical intervention rarely required [2, 4]. Most CAPs occur in the target intervention vessels [2, 3]. “Delayed” CAPs are defined as the presence of fluid in the pericardial space requiring intervention after the patient has left the catheterization laboratory but before hospital discharge post-PCI [4]. Its incidence has been reported to be only 0.04% [4], but it can be potentially lethal. Recent reports have shown that delayed CAPs usually occur after complex PCIs and are primarily attributed to distal wire perforations in the target intervention vessels [2–4, 9]. However, the occurrence of a delayed CAP in nontarget vessels has rarely been reported. A recent report demonstrated that delayed CAPs were likely caused by distal guidewire exiting smaller arteries that were often missed in the catheterization laboratory and only later identified as tamponade in the ward. However, the differences in outcomes between acute and delayed CAPs remain unclear [3]. However, because we confirmed satisfactory angiographic results without contrast enhancement with extravasation or oozing (Figure 2e,f and Supporting Information 1 and 2), we probably did not miss CAPs. Although the mechanism(s) of delayed CAP onset has not been entirely elucidated, its pathogenesis may be multifactorial. Key factors in delayed CAPs may include the potent platelet-suppressing effects of dual-antiplatelet therapy, the prolonged anticoagulant effect of heparin used during PCI, the use of stiff-tip and hydrophilic guidewires [2, 3], unintentional guidewire entry into small coronary arteries, and fragile coronary arteries associated with coronary risk factors. In this case, dual-antiplatelet therapy was administered, and some coronary risk factors included hypertension, Type 2 diabetes mellitus, and dyslipidemia. Additionally, a hydrophilic guidewire was positioned into the small coronary artery. As a precaution, we should have monitored the ACT after PCI, even with satisfactory angiographic results without contrast enhancement with extravasation or oozing. If the ACT had remained prolonged after PCI, reducing the heparin effect with a protamine adjunction could have been considered. Additionally, when protecting small side branches with guidewires, it may be better to avoid using hydrophilic guidewires. Moreover, because most delayed CAPs occur within the first 5 h after PCI [4], interventionalists should be aware of the possibility of their occurrence during this period. Further investigations are required to clarify the mechanisms underlying a delayed CAP.

A recent report demonstrated potential approaches for CAPs during PCI and highlighted different management strategies that should be considered according to the perforation site [2]. Based on these strategies, we performed intravenous administration of a high dose of saline, pericardiocentesis, balloon dilation, weakening of heparin by a protamine adjunction, and injection of autologous blood clots. However, these were ineffective in this case. Finally, intracoronary administration of gelatin microspheres [6] (Serescue, Astellas) (Figure 2l) resulted in complete occlusion of the delayed CAP site (Figures 2j, 2k, and 2l and Supporting Information 5, 6, and 7). Thus, this gelatin microsphere injection may be an effective and feasible strategy for managing delayed CAPs in small vessels. To our knowledge, this is the first report of this particular product in managing a CAP. There are several types of commercially available microspheres worldwide, and all have been reported to be useful for treating CAPs [6, 10, 11].

## 4. Conclusions

Delayed CAPs are extremely rare but can occur even in nontarget vessels and are potentially lethal. Meticulous attention to any patient signs, symptoms, a nimble definite diagnosis, and effective, timely management of delayed CAPs may be essential for practicing cardiologists to reduce subsequent complications and improve patient prognosis after PCI, especially in cases with complex lesions.

## Data Availability

The authors have nothing to report.

## Supporting Information

Supporting Information 1 Video S1: The final coronary angiographies (CAG) viewed from right caudal anterior oblique views demonstrated satisfactory angiographic results with no contrast enhancement with extravasation and/or oozing.

Supporting Information 2 Video S2: The final coronary angiographies (CAG) viewed from the left cranial anterior oblique views demonstrated satisfactory angiographic results with no contrast enhancement with extravasation and/or oozing.

Supporting Information 3 Video S3: Emergency CAG viewed from right caudal anterior oblique views revealed an Ellis Type IIIa CAP in a small distal branch of the diagonal branch (DB).

Supporting Information 4 Video S4: Emergency CAG viewed from the left cranial anterior oblique views revealed an Ellis Type IIIa CAP in a small distal branch of the diagonal branch (DB).

Supporting Information 5 Video S5: After injection of gelatin microspheres.

Supporting Information 6 Video S6: CAG viewed from left cranial oblique views revealed the perforated distal site of the DB was completely occluded.

Supporting Information 7 Video S7: Right caudal oblique views revealed the perforated distal site of the DB was completely occluded.
